# Two-Stage Prosthetic Breast Reconstruction With Integrated Versus Remote Port Expanders: A Comparison of Complication Rates

**Published:** 2019-04-17

**Authors:** Joshua H. Choo, Mitchell J. Buller, Michelle O'Brien, Ron Hazani, Adam Augenstein, John P. Tutela, Bradon J. Wilhelmi

**Affiliations:** ^a^Department of Plastic Surgery, University of Louisville School of Medicine, Louisville, Ky; ^b^Department of Plastic Surgery, University of South Florida, Tampa; ^c^Department of Orthopaedics, University of Cincinnati School of Medicine, Cincinnati, Ohio; ^d^Department of Plastic Surgery, University of Louisville School of Medicine, Louisville, Ky

**Keywords:** remote tissue expanders, integrated tissue expanders, breast reconstruction, remote port complications, prosthetic breast reconstruction

## Abstract

**Background:** Ever since their introduction, tissue expanders for breast reconstruction have undergone a gradual evolution from remote port expanders to the integrated port expanders commonly in use today. Integrated port expanders have been widely adopted because of their ease of use and reliability, and though the convenience of integrated port expanders over remote port expanders is clear, a side-by-side comparison of complications has not been performed. A same-surgeon, same-institution study was conducted comparing the complication rates of remote versus integrated tissue expanders. **Methods:** A retrospective review was conducted of 107 patients who underwent breast reconstruction with tissue expanders. Remote tissue expanders were used in 21 consecutive patients (*n* = 42) and integrated port tissue expanders in 86 consecutive patients (*n* = 128). Patients who had received prior or concurrent breast irradiation were excluded from the study. Overall complications were compared, followed by complications that were broken down according to mechanical and infectious complications. **Results:** Fisher's exact test demonstrated a statistically significant increase in the rate of overall complications in remote port expanders compared with integrated port expanders (19% vs 7%; *P* = .024). Similarly, a statistically significant difference in the rate of mechanical complications between the 2 groups was found (7% in remote vs 0.8% in integrated, *P* = .047). When the rates of infectious complications were compared between the 2 groups, however (12% in remote vs 6% in integrated), no significant difference could be found (*P* = .312). **Conclusion:** In this retrospective review of prosthetic breast reconstructions, increased overall complications were observed with remote tissue expanders that were mainly mechanical in nature. The higher rate of infection observed in the remote port group was not statistically significant. Our study shows that remote port expanders do in fact have a higher complication rate than integrated port expanders. This should be taken into account when considering the use of remote port expanders in certain clinical scenarios.

In the United States alone, an estimated 246,660 cases of invasive breast cancer in women were diagnosed in 2016.^1^ Prosthetic reconstruction has become an increasingly popular treatment option, with the percentage of women choosing this method of reconstruction climbing from 11.6% in 1998 to 36.4% in 2011.^2^ The number of breast reconstruction procedures performed annually is up 35% since 2000, with implant-based reconstruction accounting for 81% of the 106,338 procedures performed in 2015.^3^

While the introduction of skin and nipple-sparing mastectomies has permitted new methods of breast reconstruction, 2-stage reconstruction beginning with tissue expander placement is still the most common method of prosthetic breast reconstruction. Radovan was the first to describe the use of a tissue expander to allow serial volume expansion of the skin before the placement of a permanent implant. Early prototypes were smooth-walled and filled via remote ports, which made them susceptible to kinking, twisting, and leakage. Expanders with integrated ports were later introduced to eliminate these problems. Further refinements over time led to the introduction of textured surfaces to minimize capsular contracture. Ports are now larger with self-sealing peripheries to minimize the occurrence of errant needle access, and most also have suture tabs to allow for a higher degree of pocket control.

When integrated port expanders were first introduced, concerns existed over the method of access (ie, through thin and vascularly compromised mastectomy flaps) and its potential to raise infection and skin necrosis rates. There were also concerns about how the magnetic integrated ports would affect the delivery of radiation to the breast. Safety and reliability of integrated port expanders are now well-established,^4^^,^^5^ and while concerns about dose attenuation and heterogeneity do exist, most studies have shown that these effects are small and can be compensated for through heterogeneity corrections.^6^^-^8

While it may be tempting to conclude that remote port expanders have little part in current prosthetic breast reconstruction, the use of a remote port expander has its advantages in certain subsets of patients. When combining autologous and prosthetic breast reconstruction, magnetic port localization becomes less reliable as the thickness of the intervening flap increases, and a remote port expander may be a preferred alternative in these patients.^9^ The majority of expanders with integrated magnetic ports are magnetic resonance imaging (MRI)-unsafe,^10^ and patients with an anticipated need for MRI during the expansion process may therefore benefit from remote port expander placement.^9^ In addition, the Food and Drug Administration recommends caution when using integrated port expanders in patients with implantable defibrillators or pacemakers, as serious adverse interactions have been reported.^11^^-^13 In these patients, the use of remote port expanders may also be justified. In light of these considerations, it is worthwhile to review the risk profile of remote tissue expanders in comparison with integrated port expanders.

A review of the literature shows no head-to-head comparisons of remote and integrated port tissue expanders and their respective complication rates. Integrated port tissue expanders have been shown to have lower complication rates than what was historically observed with remote port expanders, but it is difficult to determine which complications were inherent to expander design. The purpose of this study was to retrospectively assess the rates of complications and infection in remote versus integrated ports in our patients undergoing 2-stage prosthetic breast reconstruction.

## METHODS

A single-center retrospective review of 107 consecutive cases of 2-stage prosthetic reconstruction with remote versus integrated tissue expanders was conducted (Table 1). These 170 expanders were placed in 107 patients undergoing 2-stage breast reconstruction (Table 2). A total of 170 expanders were placed, with 64 patients with bilateral and 44 with unilateral breast reconstruction. Remote ports (Mentor Spectrum Remote; Mentor Corporation, Santa Barbara, Calif) were used in 21 patients and all were bilateral, for a total of 42 remote port expander reconstructions. Integrated ports (Mentor CPX3; Mentor Corporation) were used in 86 patients, with 42 bilateral and 44 unilateral, for a total of 128 expander reconstructions. Patients who had prior or planned radiation were excluded. Institutional preference was the determining factor for whether a patient received a remote tissue expander versus an integrated port expander; most of the remote port tissue expanders were performed earlier in the senior surgeon's practice, reflecting the preferences of the radiation oncology department at our institution at that time. Patient records were retrospectively reviewed for instances of complications and infection.

Given the small number of complications observed (1/4 expected cell frequencies <5), a Fisher exact test was used to compare the 2 groups. Overall complications were compared, followed by complications that were categorized as either mechanical or infectious.

## RESULTS

Our review revealed a complication rate of 19% (8/42) associated with the use of remote port tissue expanders and a rate of 7% (9/128) with the use of integrated port expanders (Table 3). Five of the 8 complications associated with remote port expanders were due to infection. The remaining 3 complications were related to mechanical problems with the port that necessitated a re-operation, such as kinking and turnover of the port and port extrusion (Table 4). Eight of 9 complications in the integrated port expander group were due to infection, with the one remaining complication occurring secondary to expander deflation (mechanical). Comorbidities and other associated risk factors were examined and found not to be statistically significant between the 2 patient groups.

A Fisher exact test demonstrated that the increased rate of complications among remote port tissue expanders in immediate postmastectomy breast reconstruction was statistically significant (*P* = .024) (Table 3). Similarly, a statistically significant difference in the rate of mechanical complications between the 2 groups was found (7% in remote vs 0.8% in integrated, *P* = .047). When the rates of infectious complications were compared between the 2 groups, however (12% in remote vs 6% in integrated), no significant difference could be found (*P* = .312).

## DISCUSSION

Advantages and disadvantages exist for both the remote and integrated port expanders. Remote port expanders confer the theoretical advantage of allowing the surgeon to select the site of injection in a place that is easily palpable and away from the tissue expander and the mastectomy flaps, thereby avoiding dependence on a magnet for port location and minimizing the risk of errant needle puncture in the case of thick overlying autologous flaps. Disadvantages include patient discomfort due to the port's location in the axillary region as well as the risks of mechanical complications (Table 5).

There are several advantages of integrated port tissue expanders. Integrated tissue expander insertion allows for the use of the existing mastectomy pocket and avoids having to create a tunnel and additional pocket for the port. Integrated expanders now have tabs to secure them in the proper position to avoid lateral displacement and axillary expansion. The newer expanders also have a wide self-sealing zone around the port to minimize the risk of leakage with errant needle access. Modern integrated port tissue expanders are more anatomically shaped, with increased distension at the lower pole producing expansion in the teardrop shape of the natural breast. The texturing present in these tissue expanders has been believed to lessen the risk of capsular contracture and facilitate the expansion process. While no rigorous head-to-head comparisons have been made, these observed benefits have led to the routine use of textured integrated port expanders over remote port expanders.

Certain theoretical disadvantages of integrated port expanders include attenuation of the radiation beam. Previous reports have suggested that regions of underdosing exist in the tissue surrounding the magnet.^6^^,^^14^ More recent literature confirms the decreased dosing and increased heterogeneity of the radiation received, but the clinical significance of the small area of the underdosing remains unclear.^7^^,^^15^ Most radiation protocols can now account for dosing heterogeneity introduced by the presence of the metallic port. At this time, the general consensus is that volume of attenuation is small and poses no contraindication to the use of an integrated port tissue expander.^8^

Our study indicates an increased rate of complications among these remote port expanders when compared with integrated port expanders. These complications are primarily mechanical in nature and include flipping, kinking, or extrusion of the port, as well as expander overlap of the port. While a statistical significance was not found between the rates of infection in the 2 groups, the data trended toward an increase in infections in the remote port (5/42 patients) versus integrated port (8/128) tissue expanders.

This study has several limitations, including its retrospective nature and the single center at which it was performed. This study is also limited by the fact that the number of integrated port placements exceeds the number of remote port expansions and that other variables such as shape and texturing were not accounted for.

Some of these limitations are a consequence of the current catalogue of devices offered by the 3 major device companies. While the options for textured integrated port expanders are abundant, the choices for remote port tissue expanders are much more limited. Invariably only one option exists: a round smooth tissue expander.

The rate of capsular contracture was not compared between the 2 groups in this study, although such a comparison would have potentially been meaningful. While the lack of a statistically significant difference in infection rate is, in part, due to the small patient population surveyed and the discrepancy in the number of remote and integrated ports implanted, certain patients, including obese patients, those undergoing combined autologous/prosthetic reconstruction, and those for whom an integrated port is contraindicated may benefit from the use of remote port expanders.

## Figures and Tables

**Table 1:** Tissue expanders used

Remote port	42
Integrated port	128

**Table 2:** Types of tissue expanders used in 107 patients undergoing reconstruction

		Tissue expanders used		
	Patients	Unilateral	Bilateral	Total
Remote port	21	0	21	42
Integrated port	86	44	42	128

**Table 3:** Total complications by expander type*

Expander	Total	Total	Percentage
type	implanted	complications	of total
Remote	42	8	19
Integrated	128	9	7

*Remote port expanders show a statistically significant increase in complication rate (*P* = .024).

**Table 4:** Complications associated with the use of remote port tissue expanders

Complication	Number of patients	Percentage of patients
Infection	5	12
Tube kinking	1	2
Port turnover	1	2
Port extrusion	1	2
Total	8	19

**Table 5:** Theoretical complications of the use of remote port tissue expanders

Theoretical complications
Port turnover
Kinking and blockage of tubing
Migration of port
Tubing migration over port
Port extrusion
Port infection
Implant expansion over port
Difficulty of port placement
Difficulty of port removal
